# Tumor-Derived Microvesicles Promote Kidney Regeneration and Cytoprotective Immunomodulation

**DOI:** 10.3390/ph18101520

**Published:** 2025-10-10

**Authors:** Galina V. Seledtsova, Victor I. Seledtsov, Ayana B. Dorzhieva, Elena A. Blinova, Adas Darinskas, Elena A. Prokopyeva, Alexei A. von Delwig

**Affiliations:** 1 Institute for Fundamental and Clinical Immunology, 630099 Novosibirsk, Russia; galina-seledtsova@yandex.ru (G.V.S.); dorzhieva-ayana@yandex.ru (A.B.D.); blinovaelena@yandex.ru (E.A.B.); 2 Petrovsky National Research Centre of Surgery, 119991 Moscow, Russia; 3 JSC Innovita Research, 06118 Vilnius, Lithuania; darinskas.adas@gmail.com; 4 Institute of Medicine and Medical Technologies, Novosibirsk State University, 630090 Novosibirsk, Russia

**Keywords:** tumor, mesenchymal stem cell, microvesicle, regeneration, chronic kidney injury

## Abstract

**Background:** A comparative study was conducted to evaluate the potential of extracellular, tumor-derived microvesicles (MVs)s in promoting kidney regeneration. **Methods:** MVs were collected from L929 sarcoma, LLC, and B16 melanoma cells, and mesenchymal stem cells (MSCs). The regenerative activity of MVs was evaluated in an experimental murine model of chronic kidney injury (CKI). **Results:** Both tumor-derived MVs (T-MVs) and MSC-derived MVs (MSC-MVs) significantly improved kidney function and histological structure. Specifically, the height of collecting tubules in the middle third of the renal medulla returned to normal levels following MV treatment. Both T-MVs and MSC-MVs reduced the proportion of pro-inflammatory CD4+CD44+ T cells in renal cell infiltrates and spleens of CKI mice. Furthermore, treatment with these MVs increased the number of natural CD4+CD25+FoxP3+ regulatory T cells in the spleen, indicating their immunomodulatory effects. **Conclusions:** These findings suggest that T-MVs, similar to MSC-MVs, possess a universal capacity to promote kidney tissue regeneration and exert anti-inflammatory immunomodulatory effects.

## 1. Introduction

Extracellular vesicles (EVs) are phospholipid bilayer-enclosed particles released by all cell types. These vesicles can be easily separated from tissue culture supernatants and biological fluids, including blood, saliva, breast milk, cerebrospinal fluid, and malignant ascites. EVs can be categorized into two distinct subtypes based on their biogenesis: exosomes and microvesicles (MVs). Exosomes are formed through the inward budding of the endosome limiting membrane, leading to the creation of multivesicular bodies that fuse with the plasma membrane and are subsequently released into the extracellular space. Extracellular MVs arise from plasma membrane invagination, forming neck-like structures that eventually undergo vesicle scission. Exosomes are typically 50–150 nm in size, while MVs range from 100 to 1000 nm in diameter. Distinguishing between exosomes and MVs can be challenging due to overlapping sizes, shared surface proteins, and the absence of specific markers [1].

Cell activation and apoptosis are well-established stimuli for the formation and secretion of EVs, which serve as carriers for various biomolecules, including proteins, lipids, DNA, and different RNA species. The loading of EVs with molecular cargo occurs through an organized process that controls biomolecule sorting [1]. The primary function of EVs is to transport and protect biomolecules from degradation by extracellular enzymes and other aggressive factors. Published evidence suggests that MVs can also transport and protect cell organelles, such as mitochondria. MVs possess intrinsic stability due to their overall negatively charged surface and expression of the surface immunoglobulin CD47 biomarker, which enables evasion from engulfment by mononuclear phagocytic cells [2]. The migration direction patterns of EVs in the body are determined by the surface receptors they express [3]. Recent reports have also demonstrated the efficient crossing of various biological cell membrane barriers, including the blood–brain barrier, by EVs [1]. A low pH environment has been shown to stimulate EV fusion with cell membranes [4].

Mesenchymal stem cells (MSCs) have been extensively studied and applied in regenerative medicine, with numerous clinical trials conducted for a wide range of diseases [5]. Recently, research focus has shifted towards the investigation of EV preparations derived from MSCs. In particular, MSC-derived EVs have shown promising potential for in vivo tissue regeneration and the treatment of various diseases in experimental models, including respiratory, renal, hepatic, neurodegenerative, musculoskeletal, and cardiovascular disorders. These studies have highlighted the robust anti-inflammatory, antiapoptotic, pro-angiogenic, and immunomodulatory effects exhibited by MSC-derived EVs, similar to those displayed by MSCs in different disease models. Specifically, MSC-derived EVs from different sources have demonstrated renoprotective activity in experimental models of chronic kidney injury (CKI), diabetic nephropathy, and kidney fibrosis [6]. Administration of these EVs has been found to significantly reduce serum markers of renal injury, such as blood urea nitrogen, creatinine, and transaminase levels, while improving necrotic lesions and ameliorating pronounced signs of tubular dilatation and cast formation [7].

Currently, the treatment of CKI remains challenging and often fails to achieve the desired outcomes [5,8]. This report presents experimental data demonstrating, for the first time, that tumor cell-derived microvesicles (T-MVs) of various origins, similar to MSCs and MSC-derived microvesicles (MSC-MVs), effectively restore damaged kidney tissue and function in an experimental model of CKI. Furthermore, the observed regenerative effect may be partially attributed to the inhibition of pro-inflammatory immune reactivity.

## 2. Results

### 2.1. Size Distribution of Isolated Extracellular Vesicles

**Figure 1:**
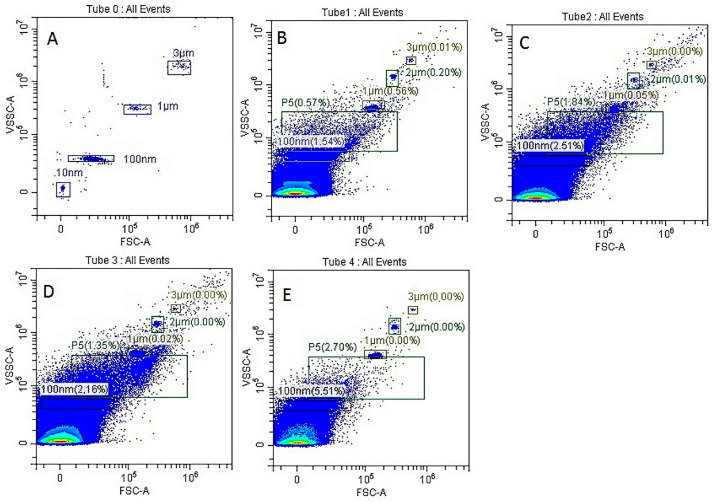
Evaluation of exovesicle sizes by flow cytometry. All exovesicles were labelled with Annexin V-FITC. (**A**) Sample without exovesicles containing standard particles labelled with Annexin V-FITC (negative control); (**B**) MSC-MVs; (**C**) B16-MVs; (**D**) LLC-MVs; and (**E**) L929-MVs.

Flow cytometry analysis revealed that the isolated extracellular vesicles were predominantly larger than 100 nm in size. This suggests that our technological approach primarily yielded extracellular MVs (Figure 1).

### 2.2. T-MVs, but Not Peripheral Blood Mononuclear Cell-Derived-MVs (PBMC-MVs), Improve the Excretory Function of Damaged Kidneys, Similar to MSCs and MSC-MVs

Creatinine is a nonenzymatic breakdown product of creatine and creatine phosphate in the body. It is cleared by the kidneys, and elevated creatinine levels typically indicate reduced filtration in renal glomeruli and decreased excretory kidney function. Fatty acid binding protein-1 (FABP1) is expressed in renal proximal tubule cells and is released into the urine in response to hypoxia caused by decreased peritubular capillary blood flow, serving as an early marker of kidney damage [9].

Our experiments demonstrated that induction of CKI resulted in a statistically significant increase (*p* < 0.001) in serum creatinine levels (from 2.41 ± 0.17 to 4.81 ± 1.75 mg/dL) and FABP1 levels (from 0.80 ± 0.21 to 1.58 ± 0.16 ng/mL). Treatment of mice with MSCs or MSC-MVs led to a reduction in blood creatinine and FABP1 levels, nearly approaching the background levels (Table 1). Importantly, administration of T-MVs derived from L929, LLC, or B16 cells exhibited similar effects on creatinine and FABP1 levels in CKI mice. No effect on kidney functionality was attributable to PBMC-MVs. This data strongly suggests that, similar to MSCs and MSC-MVs, T-MVs have the potential to restore the function of damaged kidneys, regardless of the histological origin of the T-MVs used for their generation.

### 2.3. T-MVs Improve the Histological Structure of the Damaged Kidney, Similar to MSCs and MSC-MVs

**Figure 2:**
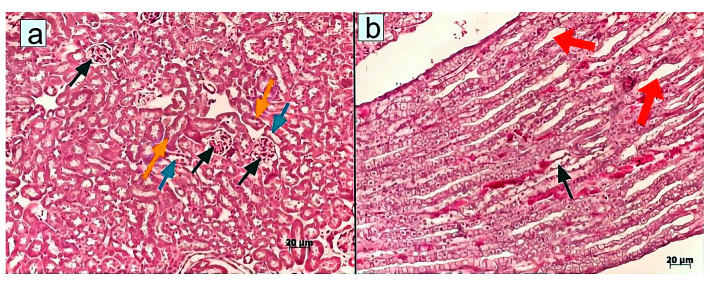
Morphological structure of renal tissue in mice with CKI: (**a**) Renal corpuscles (black arrows), proximal (yellow arrows) and distal tubules (blue arrows); (**b**) Collecting ducts (red arrows) and Henle’s loop (black arrow) in the renal medulla. Staining with hematoxylin/eosin. Induction of CKI resulted in larger diameters of renal collecting tubules, hypertrophy of cells in the collecting duct (Bellini duct), and dystrophy of cells of the Henle’s loop in the middle and lower parts of the medullary area. Morphometric measurements were taken in one field of view (ocular lens 10 × 25, objective lens 63×).

In terms of morphology, the initiation of CKI led to an increase in the size of renal collecting tubules, cellular hypertrophy in the collecting duct (Bellini duct), and cell dystrophy in the Henle’s loop within the middle and lower regions of the medullary area (Figure 2).

**Figure 3:**
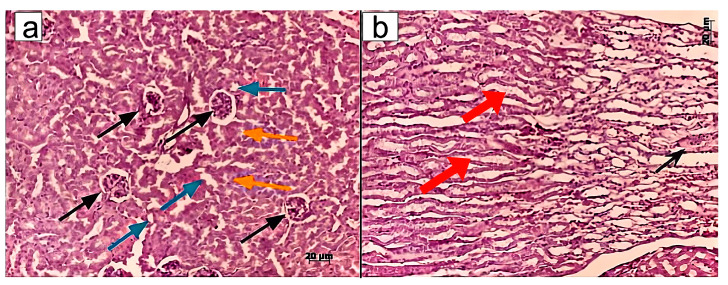
Morphological structure of renal tissue in mice with CKI treated with MSCs: (**a**) Renal corpuscles (black arrows), proximal (yellow arrows) and distal tubules (blue arrows); (**b**) Collecting ducts (red arrows) and Henle’s loop (black arrow) structures at the border between the cortex and medullary region. Staining with hematoxylin/eosin. Morphometric measurements were taken in one field of view (ocular lens 10 × 25, objective lens 63×).

**Figure 4:**
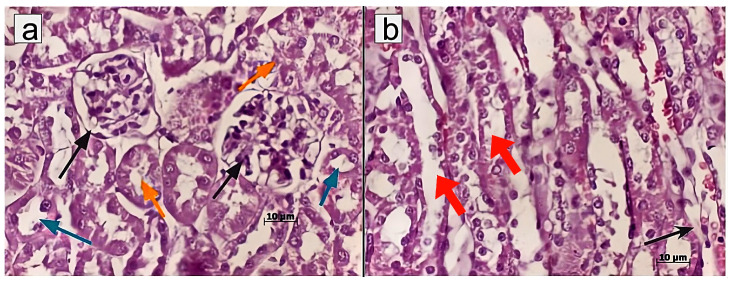
Morphological structure of renal tissue in mice with CKI treated with B16-MVs: (**a**) Renal corpuscles (black arrows), proximal (yellow arrows) and distal tubules (blue arrows); (**b**) Collecting ducts (red arrows) and Henle’s loop (black arrow) in the renal medulla. Staining with hematoxylin/eosin. Morphometric measurements were taken in one field of view (ocular lens 10 × 25, objective lens 63×).

**Figure 5:**
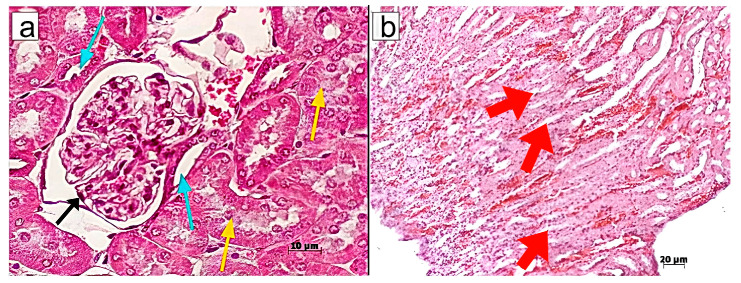
Morphological structure of renal tissue in mice with CKI treated with LLC-MVs: (**a**) Renal corpuscle (black arrow), proximal (yellow arrows) and distal tubules (blue arrows); (**b**) Collecting (Bellini) ducts (red arrows) of the renal papilla. Staining with hematoxylin/eosin. Morphometric measurements were taken in one field of view (ocular lens 10 × 25; objective lens 63×).

However, no signs of dystrophic changes in renal corpuscles were detected upon administration of MSCs or MSC-MVs into CKI-induced mice. Similar therapeutic effects were observed in mice treated with T-MVs. Specifically, morphological examination revealed well-preserved parietal and visceral layers of the renal corpuscle (Bowman’s capsule), with the urinary (Bowman’s) space clearly visible (Figure 3, Figure 4 and Figure 5).

**Figure 6:**
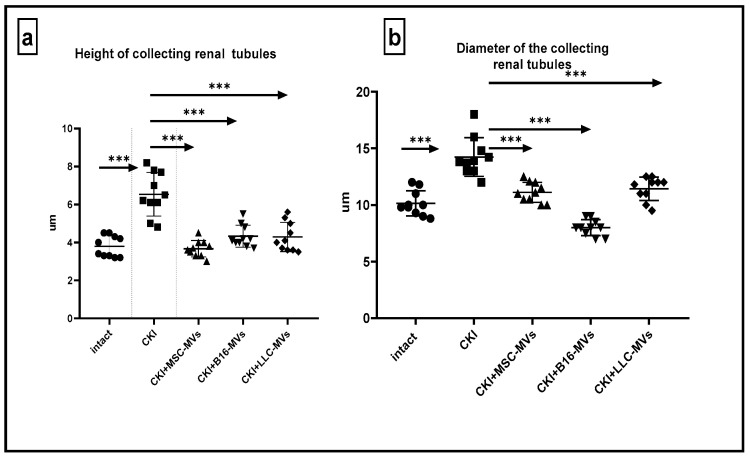
Heights of collecting tubules (µm) measured in the middle third of the renal medulla (**a**), and diameters of the collecting renal tubules (**b**) in intact and control CKI mice, as well as in CKI mice treated with MSC-MVs or T-MVs. Data were collected 11 days after administration of MVs. Statistical significance was determined using Sidák’s multiple comparisons test, n = 10, *** *p* < 0.0001.

Taken together, the treatment of CKI mice with T-MVs, as well as MSC-MVs, resulted in restoration of normal morphological structure of the damaged kidneys. The morphometric data presented in Figure 6 indicates that both T-MVs and MSC-MVs exhibited similar regenerative effects on the damaged kidney tissue.

### 2.4. T-MVs Promote Cytoprotective Immunomodulation

**Figure 7:**
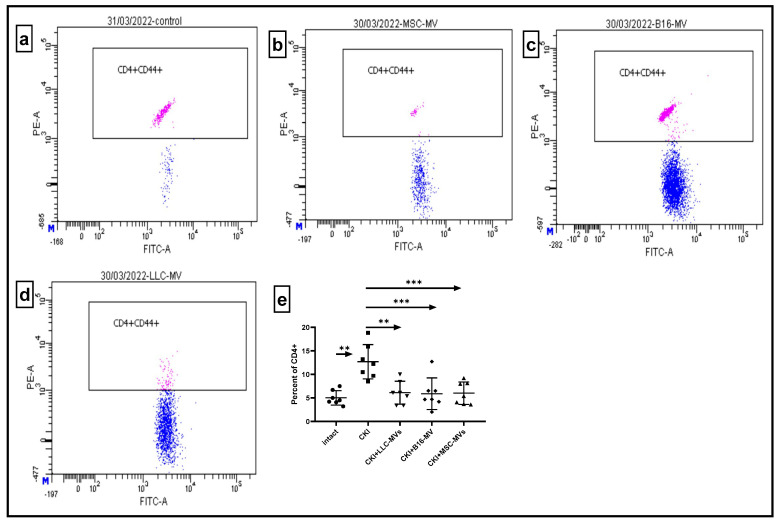
The relative levels of CD44+ T cells within the CD4+ T cell population in renal cell infiltrates: untreated CKI mice (**a**), CKI mice treated with MSC-MVs (**b**), B16-MVs (**c**), or LLC-MVs (**d**). The percentage of CD4+CD44+ T cells in intact controls, CKI mice, and MV-treated CKI mice (**e**). Data were obtained 11 days after MV administration. Statistical significance (*p*) was determined using Tukey’s multiple comparisons test, n = 6, ** *p* < 0.001, *** *p* < 0.0001.

**Figure 8:**
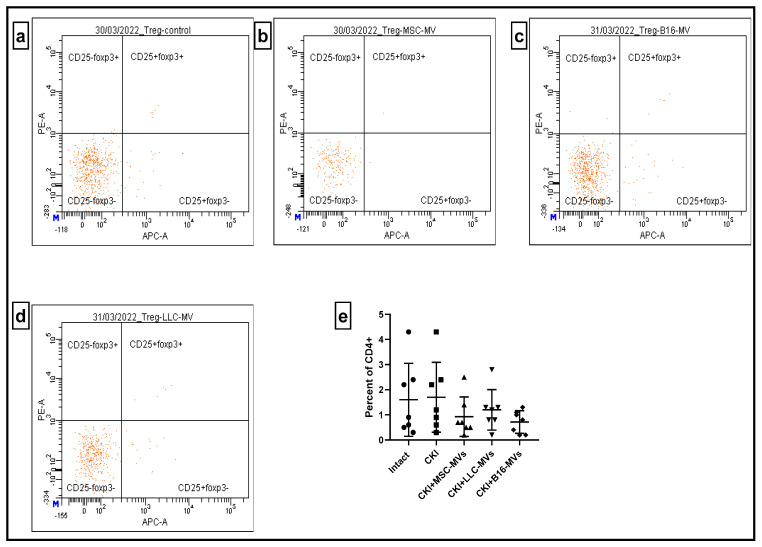
The relative levels of CD25+FoxP3+ T cells within the CD4+ T cell population in renal cell infiltrates: untreated CKI mice (**a**), CKI mice treated with MSC-MVs (**b**), B16-MVs (**c**), or LLC-MVs (**d**). The percentage of CD4+CD25+FoxP3+ T cells in untreated and MV-treated CKI mice (**e**). Data were obtained 11 days after MV administration. Statistical significance was determined using Tukey’s multiple comparisons test, n = 7.

The immune system is known to exert constant control over the regenerative processes in the body via cytodestructive and cytoprotective immune reactivity, with T cells playing a pivotal role in defining the direction and strength of such immune reactions [10]. Therefore, we studied the effect of regenerative MVs on the proportion of CD44+ and CD25+FOXP3+ T cells within the CD4+ T cell population in the renal cell infiltrates and in the spleen of CKI mice. CD44 is an adhesion molecule that functions as a receptor to glycosaminoglycan of the extracellular matrix (hyaluronan), facilitating migration and mobilization of immune cells into inflammatory loci. The expression of CD44 on T cells is typically associated with their pro-inflammatory activity, including that of pathological origin, which has been confirmed in most chronic inflammatory models of disease [11]. On the other hand, regulatory CD4+ CD25+FOXP3+ T cells possess pronounced anti-inflammatory properties, down-regulating immune-mediated cytodestructive reactions [12,13]. The data shown in Figure 7 suggests that CKI development resulted in an increased number of pro-inflammatory CD4+CD44+ T cells in renal cell infiltrates, while administration of MSC-MVs in CKI mice brought the levels of CD4+CD44+ T cells back to nearly normal values. Similar effects were observed in groups of CKI mice treated with B16-MVs or LLC-MVs. In contrast, CKI development did not significantly affect the proportion of CD4+CD25+FOXP3+ T cells in renal cell infiltrates (Figure 8), and this situation remained unchanged upon administration of MVs derived from different origins.

**Figure 9:**
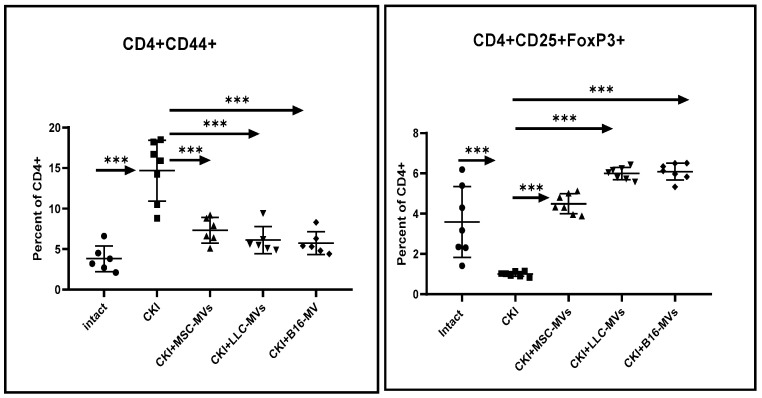
The percentages of CD44+ and CD25+FoxP3+ T cells within the CD4+ T cell population in the spleen. CKI mice were either untreated or treated with the MVs under study. Data were obtained 11 days after MV administration. Statistical significance (*p*) was determined using Tukey’s multiple comparisons test, n = 7, *** *p* < 0.0001.

Moreover, similar to the kidneys, CKI development was accompanied by an increased relative content of CD4+CD44+ T cells in the spleens (Figure 9), which returned to baseline levels upon administration of MSC-MVs or T-MVs. In contrast to the unchanged levels of CD4+CD25+FOXP3+ T cells in renal cell infiltrates in CKI mice, we observed a significant reduction in this cell population in the spleens. However, upon administration of the MVs studied here in CKI mice, the levels of cytoprotective CD25+FOXP3+ T cells practically returned to the normal values characteristic of intact animals (Figure 9).

## 3. Discussion

The tumor is known to possess pathological regenerative activity, capable of modulating the tumor microenvironment to facilitate its own growth, with tumor-derived EVs playing an important role in this process. These EVs contain a range of biologically active biomolecules that stimulate tumor growth upon entry into malignant cells. Additionally, tumor-derived EVs interact with immune cells, effectively inhibiting the development of anti-tumor cytotoxic immune reactivity and reprogramming systemic immunity to sustain tumor invasion [13]. Importantly, both tumors and normal tissues use similar molecular mechanisms to regulate regenerative processes, often involving overlapping biomolecules [14]. C. Waddington’s pioneering study suggested that innate natural regenerative mechanisms could act as regulators of cancer, reaching maximal efficiency in malignant cells [15].

In this study, we conducted a comparative analysis of T-MVs, bone marrow-derived MSCs, and MSC-MVs to evaluate their impact on regenerative processes in a murine CKI model. MSCs and MSC-derived MVs have been extensively studied in experimental and clinical settings for their potential to treat various diseases. Notably, MSC-derived MVs exhibit a regenerative profile similar to MSCs themselves [6] and have been shown to reduce reactive oxygen species (ROS) production in renal tubular epithelial cells in models of renal hypoxic injury [16]. Moreover, MSCs and MSC-derived EVs have demonstrated efficacy in reducing serum markers of kidney failure (urea, creatinine, and transaminases) and promoting kidney regeneration in experimental models of CKI, diabetic nephropathy, and renal fibrosis [7]. These effects were partly attributed to small non-coding microRNAs and transforming growth factor β, which enhance the functional activity of regulatory T cells [6].

In this study, we modeled CKI using glycerol-induced kidney injury, a well-established and reproducible model of chronic kidney disease in mice. Its principal strength is that renal damage arises directly from the nephrotoxic action of glycerol on tubular structures, rather than from secondary systemic disease. This enables a clearer interpretation of kidney-specific regenerative processes. Although no animal model can fully replicate the complexity of human CKD, glycerol-induced injury reproduces key pathophysiological features—including tubular damage, inflammation, and impaired renal function—thus providing a clinically relevant context for evaluating regenerative interventions.

Our findings strongly support the notion that T-MVs possess significant regenerative activity in the kidney, comparable or even superior to that observed with MSCs or MSC-MVs. Notably, all T-MV preparations used in this study were of non-kidney origin, suggesting that T-MVs may exhibit universal regenerative properties similar to those of MSCs and MSC-MVs. Although both extracellular MVs and exosomes contribute to regenerative activity, we propose that MVs offer superior advantages. First, MVs, like cells, express receptor molecules on their surface that enable targeted interactions with recipient cells. Second, in contrast to exosomes, MVs can transfer not only biomolecules but also cellular organelles such as mitochondria, which are typically non-viable in the extracellular space. Experimental studies have demonstrated that MVs can mediate mitochondrial transfer and transfection into target cells [17]. The transferred mitochondria enhance cellular energy production, likely contributing to the observed regenerative effects in injured organs [18].

Our experiments demonstrated that T-MVs effectively improved kidney function not only in a CKI model but also in an acute kidney injury model, where the limited time for regenerative cell growth necessitates immediate reparative mechanisms [19]. Under such conditions, we speculate that MV-mediated mitochondrial transfer provides additional energy support to the injured organ, playing a crucial role in maintaining functional activity and facilitating cellular regeneration.

The direct interaction of MVs with kidney cells plays a key role in initiating kidney regeneration [3,5,8]. Beyond their direct regenerative effects, T-MVs exhibit immunomodulatory properties similar to those of MSCs. Published data suggest that MSCs, tumor cells, and their respective extracellular MVs possess immunosuppressive activity that inhibits cytodestructive immune responses, at least in part, through the enhancement of regulatory CD4+CD25+FoxP3+ T cell function. Regulatory T cells create an immunological cytoprotective microenvironment that benefits both normal and pathological (tumor) regenerative processes [12,17]. Our study demonstrated that both MSC-MVs and T-MVs downregulated the relative content of pro-inflammatory CD4+CD44+ T cells in renal cell infiltrates and the spleens of CKI mice. Additionally, MVs restored (upregulated) the relative numbers of Tregs in the spleens of CKI mice. Although further studies are required, these data strongly suggest that immune-mediated modulation of inflammation could be one of the potential mechanisms underlying the regenerative effects of both MSC-MVs and T-MVs.

From a practical standpoint, generating MSCs is a laborious and technically demanding process requiring well-characterized donor cells. In contrast, tumor cells can be easily expanded in vitro without the need for donor material. Furthermore, T-MV isolation does not require the expensive and cumbersome ultracentrifugation or microfiltration steps necessary for exosome purification. Previous studies have demonstrated that xenogeneic MSC-derived EVs can influence the growth and functional activity of target cells [20], suggesting that therapeutic T-MV-based drugs could be sourced from both human and animal cells. MV preparations also demonstrate stability in frozen and lyophilized forms over extended periods without significant loss of specific activity [21]. These advantages make the large-scale production of T-MV-based regenerative drugs both feasible and cost-effective. Given their robust regenerative potential and immunomodulatory effects, T-MVs could fill a currently underrepresented niche in regenerative and immunotherapeutic medicine.

Despite their promising therapeutic profile, concerns remain regarding the potential oncogenic risks associated with T-MVs. Indeed, T-MVs have been shown to enhance tumor growth [22]. However, it is important to note that T-MVs do not contain inherently mutagenic substances. Instead, they may accelerate the proliferation of cells that have already undergone malignant transformation rather than directly inducing carcinogenesis in healthy cells. In our study, a six-month follow-up of mice (n = 14) treated with T-MV preparations did not reveal an increased incidence of malignancy. At necropsy, no evidence of tumor formation or neoplastic involvement of internal organs was detected. Nevertheless, the long-term safety of repeated T-MV administration, particularly in chronic disorders, warrants further investigation.

For severe conditions, such as end-stage renal failure, the potential benefits of T-MV therapy may outweigh the oncogenic risks. Given their scalability and cost-effectiveness, T-MV-based treatments could represent a transformative approach for addressing various life-threatening conditions beyond kidney disease. Importantly, our experiments demonstrated that MVs derived from histologically different tumors had comparable regenerative effects on injured kidneys. However, it is plausible that the recovery of highly specialized organ functions could be influenced by the tumor origin of the MVs. From this perspective, MVs derived from kidney cancer cells might be optimal for renal therapy, while liver diseases may benefit more from liver cancer-derived MVs, and glioma-derived MVs could be most effective for neurological disorders. This hypothesis requires further experimental validation.

As for the limitations of this study, one potential concern is the possible contamination of MV preparations with apoptotic bodies. However, we maintain that apoptotic bodies, if present, are efficiently cleared by phagocytes and degraded within minutes to hours [23], making it highly implausible that apoptotic debris could deliver sustained regenerative activity in vivo. We therefore consider their contribution negligible in the context of long-term regenerative outcomes. We emphasize that future studies aimed at a deeper characterization of MV content and purity—including the use of specific markers to distinguish apoptotic bodies from vesicles—will be essential to further strengthen the translational relevance of this approach.

Finally, our present study does not include direct mechanistic assays such as cytokine profiling or mitochondrial transfer experiments. At this stage, our conclusions are drawn from functional, morphological, and immunological outcomes rather than direct mechanistic proof. Indeed, our current research efforts are now focused on testing the mitochondrial transfer hypothesis in depth. Preliminary results are encouraging, but they remain inconclusive and raise additional questions that require careful investigation.

## 4. Materials and Methods

### 4.1. Mice

All experiments were conducted on male CBA mice aged between 4 and 6 months. The mice were provided with autoclaved food and boiled water. All procedures involving animals complied with the legislation of the Russian Federation and Directive 2010/63/EU of the European Parliament and Council of 22 September 2010 on the protection of animals used in scientific research. Euthanasia was performed using cervical dislocation.

### 4.2. Generation of Bone Marrow-Derived Mesenchymal Stromal Cells

Bone marrow was extracted from intact mice’s femoral and tibial bones using a glass homogenizer. The cells were then resuspended in cold RPMI 1640 medium. After washing, the cells were cultured in plastic flasks using a complete cell culture medium consisting of RPMI 1640 medium supplemented with 10% foetal calf serum, 2 mM L-glutamine, and antibiotics. All reagents were sourced from Sigma-Aldrich (St. Louis, MI, USA). Non-adherent cells were progressively removed starting from day 3. The spindle-shaped, plastic-adherent MSCs displayed a classical fibroblast-like phenotype and formed a complete monolayer by week 4. The MSCs were collected using a 0.25% versine–trypsin solution, followed by two washes in serum-free medium.

### 4.3. Separation of Peripheral Blood Mononuclear Cells (PBMCs)

PBMCs were isolated from blood using Ficoll–diatrizoate solution (density: 1.077 g/mL) gradient centrifugation at 1500 revolutions per minute for 40 min. Floating cells were collected, washed, counted, and utilised in subsequent experiments.

### 4.4. Tumor Cell Lines

The L929 murine fibroblast cell line, Lewis lung epidermoid carcinoma (LLC), and B16 melanoma cell lines were maintained in RPMI 1640 medium supplemented with 10% FCS, 2 mM L-glutamine, and antibiotics.

### 4.5. Generation, Isolation and Characterisation of Extracellular MVs

Apoptosis of MSCs, L929, LLC, B16 cells or PBMCs (1 × 10^6^/mL) was induced by cultivating them under conditions of oxygen deprivation in serum-free medium for 24–48 h. After cultivation, cell debris was removed by centrifugation at 2000× *g* for 20 min, and the supernatants were subjected to a second centrifugation step at 12,000× *g* for 60 min at 4 °C. The MV pellet was resuspended in 100 μL of saline, and the size of Annexin V-positive MVs was determined by flow cytometry using a CytoFlex benchtop flow cytometer (Beckman Coulter Life Sciences, Indianapolis, IN, USA) with TruCOUNT nanoparticles (TC) for bead size calibration, strictly following the manufacturer’s instructions.

### 4.6. Induction of Chronic Kidney Injury

We induced rhabdomyolysis using glycerol, administered as a 50% glycerol solution intramuscularly into the hind limb muscles at a dose of 100 µL/mouse three times at weekly intervals. Glycerol-induced rhabdomyolysis exerted a mixed influence (ischemic, toxic, and retentional) on the kidneys, predominantly damaging the epithelium of the proximal convoluted tubules [24,25,26,27].

### 4.7. Treatment of CKI Mice with MSCs or MVs

MSCs (2 × 10^6^/mouse) or MVs (derived from 2 × 10^6^ cells or approximately 50–100 µg/mouse) were injected intravenously into the tail veins of the experimental mice 3 weeks after the last glycerol administration. The experiments were terminated 11 days after the administration of MSC/MV preparations. The study included seven groups, each consisting of ten male mice:

1. Control group (CKI mice without treatment).
2. CKI group treated with MSCs.
3. CKI group treated with MSC-MVs.
4. CKI group treated with L929 cell-derived MVs (L929-MVs).
5. CKI group treated with LLC-MVs.
6. CKI group treated with B16-MVs.
7. CKI group treated with PBMC-MVs

At least two independent identical experiments were performed.

### 4.8. Biochemical Analysis

Blood creatinine levels were measured in mg/dL using the Creatinine Parameter Assay Kit (R&D Systems, Minneapolis, MN, USA). Fatty acid binding protein-1 (FABP1) levels in the blood (ng/mL) were determined using the Mouse/Rat FABP1/L-FABP Quantikine^®^ ELISA (R&D Systems, Minneapolis, MN, USA). Optical density units were converted to quantitative values using the calibration curve provided on the manufacturer’s website.

### 4.9. Histological Examinations

Murine kidneys were fixed in a 4% formalin solution, followed by standard steps of tissue processing for histopathology, including dehydration and paraffin embedding. Paraffin blocks were cut into 4–5 μm thick sections using a Rotary Microtome (Microm HM 340E; Carl Zeiss, Oberkochen, Germany) and stained with haematoxylin/eosin, Sirius red, or according to Mallory’s trichrome staining protocol. Light microscopy and microphotography were performed using a light microscope, Axioskop 40 (Carl Zeiss, Germany). Morphometric analysis of the histological kidney structure was conducted on paraffin sections by measuring the following morphometric parameters: diameters of superficial renal glomeruli, diameters of renal collecting tubules, and the size of cells in the middle third of the medullary region. Morphometric measurements were taken in one field of view (ocular lens, 10 × 25; objective lens 63×).

### 4.10. Flow Cytometry

Murine kidneys or spleens were cut into small pieces using scissors, followed by gentle homogenization in cold Versene solution using a glass homogenizer. The cell suspension was allowed to settle to remove large cell aggregates, after which density gradient centrifugation was performed in a Ficoll–diatrizoate solution (density: −1.082). After centrifugation, cells were collected, thoroughly washed, and counted. Flow cytometry analysis was conducted using a BD FACSCanto™ II Flow Cytometry System (BD International, Heidelberg, Germany) or CytoFLEX (Beckman Coulter, IN, USA) Flow cytometers with anti-murine CD4-FITC, CD44-PE, CD4-APC, CD25-FITC, and FoxP3-PE antibodies (BD Biosciences, San Jose, CA, USA), following the manufacturer’s instructions.

### 4.11. Statistics

Data analysis was performed using GraphPad Prism 8 and one-way ANOVA. Statistical significance of differences in biochemical parameters (creatinine and FABP1) and flow cytometry data (CD4+CD25+FoxP3+ and CD4+CD44+ cells) was assessed using Tukey’s multiple comparisons test, following confirmation of normality by the Shapiro–Wilk test. Outliers were identified and removed using the “Identify Outliers” function in GraphPad Prism 8 (ROUT, Q = 1%). For morphometric analyses, Sidak’s multiple comparisons test was applied. Data are presented as mean ± standard error of the mean (Mean ± SEM). The number of samples (n) analyzed is indicated in the figure legends. A *p*-value of <0.05 was considered statistically significant.

## 5. Conclusions

Our findings indicate that T-MVs, like MSC-MVs, effectively promote kidney regeneration in CKI. Unlike PBMC-MVs, which showed no regenerative potential, T-MVs demonstrated consistent therapeutic effects regardless of tumor origin. While the exact mechanisms remain to be fully defined, their regenerative activity may involve multiple factors, including mitochondrial transfer, bioactive molecule delivery, and immunomodulation. These results highlight T-MVs as a promising candidate for regenerative therapies, warranting further investigation into their clinical application and long-term safety.

The data presented here are not intended to provide an exhaustive dissection of the mechanisms underlying the regenerative activity of tumor-derived MVs, but rather to serve as a functional proof-of-concept and to map the direction for future targeted studies. Detailed analyses of cytokine networks, membrane molecules, antibody responses, and other biological mediators are indeed crucial next steps, and these will be the subject of future dedicated investigations.

## Figures and Tables

**Table 1:** Serum concentrations of creatinine (mg/dL) and FABP1 (ng/mL) in CKI mice.

Treatment	Parameter	
	Creatinine	FABP1
Intact control	2.41 ± 0.17 *^p^* ^< 0.001^	0,80 ± 0.21 *^p^* ^= 0.008^
CKI	4.81 ± 1.75	1.58 ± 0.16
CKI +MSCs	3.18 ± 0.28 *^p^* ^< 0.0002^	0.63 ± 0.16 *^p^* ^= 0.001^
CKI +MSC-MVs	2.76 ± 0.26 *^p^* ^< 0.0001^	0.78 ± 0.08 *^p^* ^= 0.008^
CKI +L929-MVs	2.26 ± 0.34 *^p^* ^< 0.0001^	0.78 ± 0.10 *^p^* ^= 0.008^
CKI +LLC-MVs	2.10 ± 0.14 *^p^* ^< 0.0001^	0.79 ± 0.11 *^p^* ^= 0.009^
CKI +B16-MVs	2.09 ± 0.12 *^p^* ^< 0.0001^	0.78 ± 0.08 *^p^* ^= 0.008^
CKI +PBMC-MVs	4.66 ± 0.24	1.11 ± 0.20

Note: The data are presented as mean ± standard error of the mean (M ± SEM). Statistical significance was assessed using Tukey’s multiple comparisons test. *p* indicates a statistically significant difference compared to the CKI group.

## Data Availability

The original contributions presented in this study are included in the article. Further inquiries can be directed to the corresponding author.
